# Sonic hedgehog signalling inhibits palatogenesis and arrests tooth development in a mouse model of the nevoid basal cell carcinoma syndrome

**DOI:** 10.1016/j.ydbio.2009.04.021

**Published:** 2009-07-01

**Authors:** Martyn T. Cobourne, Guilherme M. Xavier, Michael Depew, Louise Hagan, Jane Sealby, Zoe Webster, Paul T. Sharpe

**Affiliations:** a Department of Craniofacial Development and Orthodontics, Dental Institute, King's College London, Floor 27, Guy's Hospital, London SE1 9RT, UK; b Department of Craniofacial Development, Dental Institute, King's College London, Floor 27, Guy's Hospital, London SE19RT, UK; c Embryonic Stem Cell Facility, MRC Clinical Sciences Centre, Imperial College London, Hammersmith Hospital, London W12 0NN, UK; d Biomedical Research Centre, Guy's and St Thomas' NHS Foundation Trust, Guy's Hospital, UK

**Keywords:** Sonic hedgehog, Nevoid basal cell carcinoma syndrome, Cleft palate, Hypertelorism, Hypodontia, Craniofacial development, Ptch1, Gli1, Transgenic, Keratin-14

## Abstract

Nevoid basal cell carcinoma syndrome (NBCCS) is an autosomal dominant or spontaneous disorder characterized by multiple cutaneous basal cell carcinomas, odontogenic keratocysts, skeletal anomalies and facial dysmorphology, including cleft lip and palate. Causative mutations for NBCCS occur in the *PTCH1* gene on chromosome 9q22.3–q31, which encodes the principle receptor for the Hedgehog signalling pathway. We have investigated the molecular basis of craniofacial defects seen in NBCCS using a transgenic mouse model expressing *Shh* in basal epithelium under a Keratin-14 promoter. These mice have an absence of flat bones within the skull vault, hypertelorism, open-bite malocclusion, cleft palate and arrested tooth development. Significantly, increased Hedgehog signal transduction in these mice can influence cell fate within the craniofacial region. In medial edge epithelium of the palate, Shh activity prevents apoptosis and subsequent palatal shelf fusion. In contrast, high levels of Shh in odontogenic epithelium arrests tooth development at the bud stage, secondary to a lack of cell proliferation in this region. These findings illustrate the importance of appropriately regulated Hedgehog signalling during early craniofacial development and demonstrate that oro-facial clefting and hypodontia seen in NBCCS can occur as a direct consequence of increased Shh signal activity within embryonic epithelial tissues.

## Introduction

The nevoid basal cell carcinoma (Gorlin–Goltz) syndrome (NBCCS [MIM #109400]) (Gorlin and Goltz, 1960) is an autosomal dominant or spontaneous disorder characterized by multiple basal cell carcinomas (BCC) and epidermal cysts affecting the skin, medulloblastoma, multiple and recurrent odontogenic keratocysts of the jaws, palmar and plantar pits, dural calcification, spine and rib anomalies, and craniofacial defects (Evans et al., 1993; Gorlin, 1995; Kimonis et al., 1997; Shanley et al., 1994). Basal cell carcinomas in NBCCS are numerous and slow-growing, generally appearing from puberty onwards and rarely metastasize. In contrast, medulloblastoma normally arises during early childhood and requires immediate surgery in combination with radiation or chemotherapy. However, the five year survival rate is only around 50% and the consequences of these necessary therapeutic interventions can be devastating in the developing child (Rossi et al., 2008).

The gene mutated in NBCCS maps to chromosome 9q22.3–q31 and has been identified as *PATCHED-1* (*PTCH1* [MIM #601309]) (Hahn et al., 1996; Johnson et al., 1996). *PTCH1* is the human homologue of the *Drosophila* segment polarity gene *patched*, which encodes a twelve-pass multispan transmembrane-domain tumour-suppressor protein that contains a sterol-sensing domain (Martin et al., 2001; Strutt et al., 2001), demonstrates extensive homology to the Neimann–Pick disease (NPC1 [MIM #257220]) protein (Carstea et al., 1997; Loftus et al., 1997) and is partially related to the bacterial RND family of small molecule membrane pumps (Davies et al., 2000). In vertebrates, the predominant role of Ptch1 is thought to be as a receptor and transcriptional target within the Hedgehog signalling pathway (Goodrich et al., 1996) and in particular, for the ligand Sonic hedgehog (Shh).

Shh represents an important secreted signalling molecule that can act at both short and long-range in a variety of vertebrate organisms. Shh is essential for normal development of many regions in the embryo, in addition to subsequent homeostasis of multiple tissue lineages in the adult (Ingham and McMahon, 2001; McMahon et al., 2003). Signalling is mediated in target cells by binding of ligand to Ptch1 (Goodrich et al., 1996), an interaction facilitated by several other negatively regulated membrane-associated proteins, including Cdo, Boc and Gas1 (Allen et al., 2007; Martinelli and Fan, 2007; Seppala et al., 2007; Tenzen et al., 2006); whilst a further transmembrane protein, Hip1 is able to sequester ligand and attenuate signalling (Chuang and McMahon, 1999). Paradoxically, in the absence of ligand Ptch1 inhibits the activity of Smoothened (Smo) (Stone et al., 1996; Taipale et al., 2002), a seven-pass multispan transmembrane-domain protein absolutely required for intracellular transduction (Zhang et al., 2001). Binding of Shh derepresses Smo function and allows pathway activation, although as a direct transcriptional target of signalling, Ptch1-mediated sequestration and degradation of Shh rapidly inhibits pathway activity in responding cells (Casali and Struhl, 2004; Chen and Struhl, 1996). This relative buffering by Ptch1 influences both the concentration and duration of signal activity in determining the cellular response (Casali and Struhl, 2004; Dessaud et al., 2007). Within the cell, vertebrate Hedgehog signalling is mediated through the modification of Gli protein transcriptional activity (Bai et al., 2002, 2004; Mo et al., 1997; Park et al., 2000; Stamataki et al., 2005). Primarily, this occurs by preventing degradation of Gli2 (and Gli1) transcriptional activators (Pan et al., 2006) and promoting suitable processing of the Gli3 transcriptional repressor (Tempe et al., 2006; Wang and Li, 2006). More recently, it has become clear that normal Shh function also requires the primary cilium (Huangfu and Anderson, 2005; Huangfu et al., 2003) and that fine control of Gli protein transcriptional activity takes place within this organelle; a process dependent upon normal intra-flagellar transport (Corbit et al., 2005; Haycraft et al., 2005; Liu et al., 2005; May et al., 2005; Rohatgi et al., 2007). The majority of germline *PTCH1* mutations in NBCCS are nonsense or frameshift and lead to the synthesis of a truncated protein (Lindstrom et al., 2006), with haploinsufficiency thought to form the basis of the developmental abnormalities (Wicking et al., 1997). In the absence of normal Ptch1 function, regulation of Hedgehog signalling is compromised (Goodrich et al., 1997) with a failure to repress Smo leading to cell autonomous activation in a ligand-independent manner, increasing both the range and duration of the effective signal (Chen and Struhl, 1996). Together, these consequences of reduced PTCH1 function lead to a marked increase in Hedgehog signal activity within affected target cells and this is thought to be the developmental basis of NBCCS.

The craniofacial anomalies described in association with NBCCS include macrocephaly, frontal and parietal bossing, broad nasal root, ocular hypertelorism and mandibular prognathia; which together produce a characteristic facies. In addition there is a significantly increased incidence of cleft lip and palate (Evans et al., 1993; Shanley et al., 1994). Currently, little is known regarding the molecular basis of these malformations affecting the head and face, although Shh is known to play an important role during early craniofacial development. In particular, a loss of signalling has been associated with holoprosencephaly, cleft palate and disrupted tooth morphogenesis (Chiang et al., 1996; Dassule et al., 2000; Gritli-Linde et al., 2002; Rice et al., 2004; Seppala et al., 2007); whilst increased signalling in the early facial processes can lead to hypertelorism (Hu and Helms, 1999). Here we have investigated the craniofacial phenotype of NBCCS using a previously described transgenic mouse model that expresses *Shh* in basal epithelium from the early stages of embryogenesis (Adolphe et al., 2004; Oro et al., 1997). In particular, increased signalling in the early maxillary processes causes cleft palate, preventing fusion of the secondary palatal shelves; whilst in both incisor and molar tooth germs, odontogenesis arrests at the bud stage. These mice provide evidence that in addition to the epidermis, inappropriate regulation of Shh signal activity in epithelial tissues of the craniofacial region is an important determinant of the NBCCS phenotypic spectrum.

## Materials and methods

### Generation of K14-*Shh* transgenic mice

A DNA construct incorporating the complete open reading frame of mouse *Shh* cloned downstream of a human Keratin-14 (K14) promoter was obtained as a kind gift from Brandon Wainwright (University of Queensland). The human K14 promoter can drive gene expression in stratified squamous epithelia of transgenic mice (Vassar et al., 1989) and this includes oral and dental epithelium of the maxilla and mandible from E11.75 (Dassule et al., 2000). The K14-*Shh* transgene was isolated following an EcoRI/HindIII double digest and pronuclear injection of DNA performed on embryonic (E) 0.5 CBA/C56 BL6 embryos. Injected embryos were transferred into the uteri of pseudopregnant female CBA/C56 BL6 recipients and harvested at embryonic stages ranging from E13.5–E17.5, following maternal sacrifice with cervical dislocation. The embryonic phenotype of these transgenic mice is associated with perinatal lethality, which precludes establishment of a line (Adolphe et al., 2004). Transgenic progeny were identified using PCR analysis of tail snip DNA isolated using a GenElute^™^ mammalian genomic DNA miniprep kit (Sigma Aldrich) and amplified with human K14-specific primers (F: 5′-TCT CGC CTC TCT CTG GTC AT-3′ and R: 5′-CCT GAT ACA CAA AAA CAT CAG GA-3′). These primers generate a 328 bp fragment under the following conditions: 94 °C 2 min, 94 °C 45 s, 50 °C 45 s, 72 °C 45 s for 30 cycles (Adolphe et al., 2004). A total of 42 transgenic embryos were identified from a total of 238 produced, which gave an 18% success rate following pronuclear injection and maternal transfer.

### Histological and skeletal analysis

For histological analysis, embryos were fixed in 4% paraformaldehyde (PFA) at 4 °C, dehydrated through a graded ethanol series, embedded in paraffin wax, sectioned at 7 μm and stained with haematoxylin and eosin. Differential staining of bone and cartilage was carried out on E17.5 mice fixed overnight in 95% ethanol then skinned and eviscerated. Cartilage staining was achieved by soaking in a solution of 76% ethanol, 20% glacial acetic acid and 0.015% alcian blue 8GX (Sigma Aldrich) for 24 h, differentiating for 7 days in 95% ethanol, macerating in 1% KOH for 24 h and then washing overnight under running tap water. Bone staining was carried out by subsequent immersion in 0.1% aqueous alizarin red S (Sigma Aldrich), with the addition of several drops 1% KOH to enhance darkness of the red. Samples were washed for 30 min under running tap water, decolorized in 20% glycerol in 1% KOH for 1–2 weeks and prepared in increasing concentrations of glycerol in 70% ethanol to a final concentration of 100% glycerol. Skeletal preparations were photographed in light-field, submerged in 100% glycerol using a Leica stereomicroscope.

### Explant culture

For explant culture, palatal shelves (E13.5) and developing mandibles (E12.5 and E13.5) were dissected under a stereomicroscope and placed on 0.1 μm Millipore filters on 0.25 mm diameter stainless steel wire mesh in a Falcon organ culture dish containing DMEM (Sigma Aldrich), 10% fetal calf serum and 20 U/ml penicillin/streptomycin. For palatal shelves, the medial edges were orientated in contact with each other on the filter (Brunet et al., 1993). After a 72 h period of culture in an air incubator at 37 °C, explants were either prepared for proliferation assay (see below) or fixed directly in 4% PFA for 24 h at 4 °C overnight and then dehydrated through a graded series of ethanols, embedded in paraffin wax and sectioned at 7 μm.

### Proliferation assay

Assays for cell proliferation were carried out using a Zymed BrdU Labelling and Detection Kit (Invitrogen). Both palatal and mandibular cultures were labelled with BrdU-labelling reagent (Invitrogen) diluted 1:100 in culture medium 2 h prior to fixation. Embryos were fixed in Carnoy's fixative at 4 °C overnight, dehydrated in methanol, embedded in paraffin wax and sectioned at 7 μm. For the palatal shelf cultures, the percentage of BrdU-positive cells were calculated after blind counting by one individual on two separate occasions one week apart and the mean value taken. Left and right explants from a total of 4 WT and 4 K14-*Shh* mice were analyzed, which included a total of 32 WT and 28 mutant sections. BrdU-positive cells were counted within the epithelium and mesenchyme of the anterior and posterior palate using an ocular scale grid. Specifically, this covered an area of mesenchyme 0.013 mm^2^ bounded by a 0.2 mm length of epithelium. Student's *t*-test was used to analyze the significance of the difference in the rates of BrdU incorporation and a *P* value less than 0.05 was considered statistically significant.

### In situ hybridisation

Section radioactive in situ hybridisation was carried out as previously described (Cobourne et al., 2004). Light and dark-field images of sections were photographed using a Zeiss Axioscop microscope and merged in Adobe photoshop CS.

### Apoptosis

Immunohistochemical detection of apoptotic cell death was carried out using Terminal deoxynucleotidyl transferase-mediated dUTP Nick End Labeling (TUNEL). Briefly, tissues were fixed overnight in 4% PFA at 4 °C, embedded in paraffin wax and sectioned at 7 μm. TUNEL was carried out using an In Situ Cell Death Detection Kit (Roche Diagnostics) according to the manufacturer's instructions.

### Scanning electron microscopy

For scanning electron microscopy, tissues were fixed and stored in 2.5% glutaraldehyde in 0.1 M sodium cacodylate buffer, rinsed in 0.1M cacodylate buffer and postfixed in 1% osmium tetroxide in water for 90 min. This was followed by dehydration through a graded series of acetone in water, critical point drying in liquid CO_2_ and sputter coating with gold. Tissues were examined and recorded in a Phillips SEM501B scanning electron microscope fitted with a Deben Pixie digital scan generator and recorder.

## Results

### Multiple defects in K14-*Shh* mice phenocopy NBCCS

**Fig. 1:**
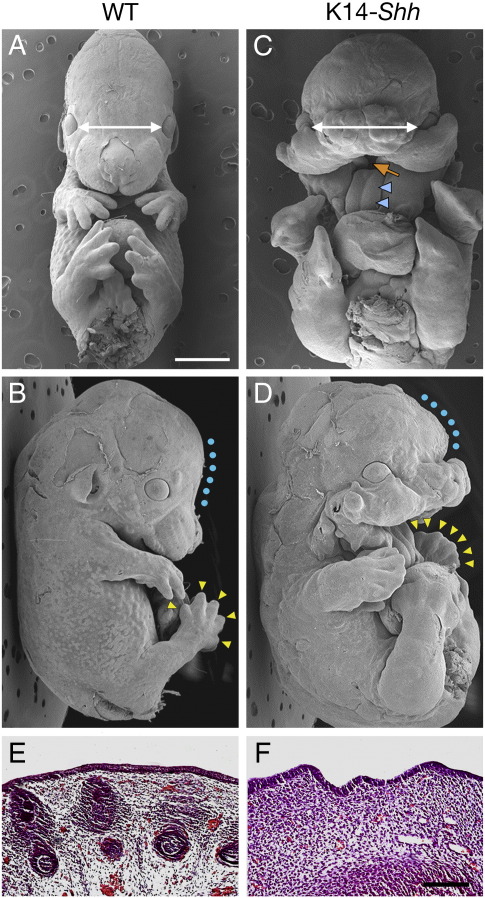
Developmental defects in K14-*Shh* mice. (A–D) Comparison of E16.5 WT and K14-*Shh* embryos using scanning electron microscopy. (A, B) WT and (C, D) K14-*Shh* embryos in frontal (upper panels) and profile (lower panels) view. K14-*Shh* embryos were larger than their WT littermates and a number of anomalies affecting the craniofacial region were present, including prominence of the frontal region (compare blue dots), hypertelorism (compare white double arrows), cleft palate (orange arrow) skeletal open bite and glossoptosis (blue arrowheads). In addition, the limbs were truncated and there was both hard and soft tissue polysyndactyly (compare yellow arrowheads). Histological analysis of WT and K14-*Shh* skin at E16.5. (E) WT and (F) K14-*Shh* mutant. The mutant skin was characterized by fragility and an epidermis lacking any hair follicles or sebaceous glands. Scale bar in A = 1 mm for A–D and in F = 50 μm for E, F.

The spectrum of developmental anomalies seen in NBCCS is reflected in the phenotype of K14-*Shh* mice and consistent with previous observations that overproduction of *Shh* mimics loss of Ptch1 function (Adolphe et al., 2004; Oro et al., 1997). In particular, these mice have defects that affect the skeletal tissues and skin with varying severity and include proximal–distal truncation and polysyndactyly affecting both fore and hindlimbs; ectopic cartilagenous and bony ossifications between the digits and soft tissue syndactyly. In addition, there is spina bifida, absence of the vertebral spinal processes, bifid sternum and tail kinking. The skin of K14-*Shh* transgenic mice is characterized by multiple BCC-like proliferations, associated with two distinct cellular phenotypes within the epidermis, which range from marked progenitor-cell hyperplasia and wrinkled, blistered skin through to a complete loss of tissue renewal within the epidermis and taut, shiny, translucent skin. This latter phenotype has been associated with the most severe developmental defects, including those affecting the craniofacial region (Adolphe et al., 2004). We found that a number of transgenic K14-*Shh* embryos (*n* = 34/42) demonstrated a severe craniofacial phenotype, characterized primarily by a prominence of the frontal region, hypertelorism, open-bite malocclusion and cleft palate (Figs. 1A–D and Table 1) and this was seen in association with a translucent skin phenotype (Figs. 1E, F).

**Fig. 2:**
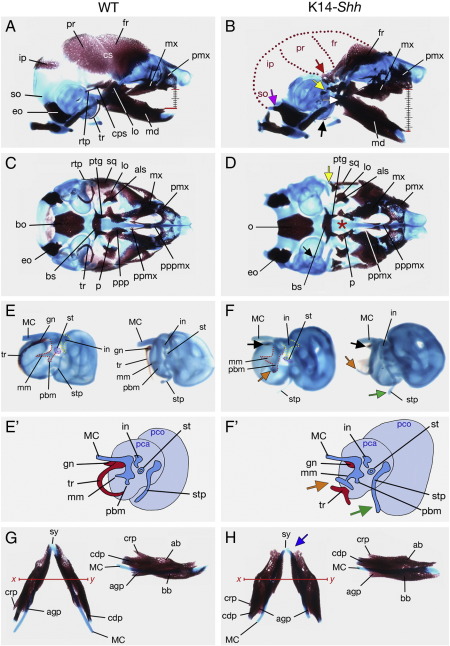
Comparison of E17.5 WT and K14-*Shh* skulls differentially stained for bone (alizarin red) and cartilage (alcian blue). (A, B) Norma lateralis. The mutant skull phenotype was dominated by an absence of membrane bones within the cranial vault (outlined with purple dots) and marked skeletal open bite (compare ruled lines). White arrow indicates ectopic dermal bones laterally adjacent to the mandibular condylar process; purple arrow indicates enlarged and poorly directed paraoccipital process of the exoccipital bone; red arrow indicates ectopic region of the frontal bone; yellow arrow indicates absent retrotympanic process of the squamosal bone. (C, D) Norma basalis. There was truncation of the premaxilla and maxilla and a cleft secondary palate associated with hypoplasia of the palatine processes of the maxilla and an absence of the palatine processes of the palatine (red ⁎). (E, F) Otic capsule and middle ear in lateral (left side) and antero-posterior (right side) views (lateral view illustrated schematically in E′, F′). Orange arrow indicates ectopic cartilage articulating with an elongated manubrium of the malleus; black arrow indicates hypoplastic gonial and ectotympanic ring; green arrow indicates partially duplicated and elongated styloid process. The malleus, incus and stapes are outlined respectively with red, yellow and purple dots in (E, F). (G, H) Occlusal (left side) and lateral (right side) views of the mandible. Note the increased bone thickness of the mutant mandibular body along the occlusal plane (*x*–*y*). Blue arrow indicates a lack of bone in the mandibular symphysis of the mutant. als, alisphenoid; bb, basal bone; bo, basioccipital; bs, basisphenoid; cps, caudal process squamosal; cdp, condylar process; crp, coronoid process; cs, coronal suture; eo, exoccipital; fr, frontal; gn, gonial; in, incus; ip, interparietal; lo, lamina obturans; MC, Meckel's cartilage; md, mandible; mm, manubrium malleus; mx, maxilla; p, palatine; pbm, processus brevis malleus; pca, pars canalicularis; pco, pars cochlearis; pmx, premaxilla; ppmx, palatal process maxilla; ppp, palatal process palatine; pppmx, palatal process of premaxilla; pr, parietal; ptg, pterygoid; rtp, retrotympanic process; so, supraoccipital; st, stapes; sq, squamosal; stp, styloid process; sy, symphysis; tr, ectotympanic ring.

NBCCS can also present with congenital anomalies of the skull and face (Kimonis et al., 1997) but to date, the aetiological basis of these defects has received little attention. We therefore analyzed the severe craniofacial phenotype of K14-*Shh* mutant mice in detail using skeletal preparation at E17.5 (*n* = 3). The most striking feature of the mutant skulls in comparison to WT was the absence of calvarial bony elements. In particular, the endochondral supraoccipital and the intramembranous interparietal and parietal bones were missing in their entirety, as was much of the caudal frontal bone (Figs. 2A, B). Numerous other skeletal alterations were evident in the neurocranium and viscerocranium. Both the basioccipital and exoccipital bones were present, although the latter was enlarged and associated with a poorly directed paraoccipital process, whilst the otic capsule was broader in both the pars canalicularis and pars cochlearis (Figs. 2C, D, E, F). A number of ectopic cartilages were seen within the otic capsule and defects identifiable within the mutant middle ear. Whilst all three middle ear ossicles were present, the manubrium of the malleus was elongated and articulated with an ectopic cartilage, which likely represented a partial duplication of the stylohyal portion of the styloid process, itself slightly elongated. Within the dermatocranial viscerocranium, the gonial and ectotympanic ring were markedly hypoplastic, the latter typically bent (Figs. 2E, F and schematic in E′, F′). The body of the squamosal bone was hypoplastic and lacked substantial retrotympanic and capsular processes. Broader premaxillae and maxillary bones were both present, though a bilateral cleft of the secondary palate was identifiable in the midline. In association with this cleft, the palatine processes of the maxillae were diminutive. In contrast, the palatal shelves associated with the hypoplastic palatine bones were absent (see Figs. 2C, D). In the dentary there was a lack of bone at the distal midline and a diminutive coronoid process, with a backward rotation of the body contributing to the marked anterior open bite (Figs. 2G, H). Ectopic dermal bones were also present, situated lateral to the mandibular condyles (see Figs. 2A, B).

### K14-*Shh* mice have cleft palate

**Fig. 3:**
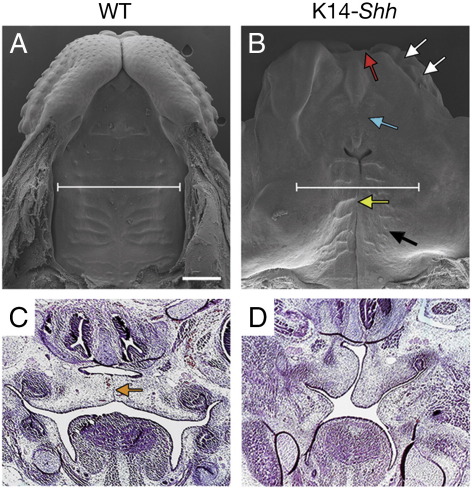
Cleft palate in K14-*Shh* mice. (A, B) Scanning electron microscopy demonstrates normal palatal closure and upper lip development at E15.5 in WT mice (A). In K14-*Shh* mutants at the same stage there is hypoplasia (red arrow) and dysmorphology (white arrows) of the upper lip, a narrow cleft of the secondary palate (yellow arrow), absence of ante-molar rugae (blue arrow) and hypoplasia of the inter-molar rugae (black arrow) (B). (C, D) Histological analysis showing fusion of the palatal shelves and degeneration of the MES (orange arrow) at E15.5 in WT mice (C). In contrast, the palatal shelves of the mutant have achieved a position above the tongue and approximated towards each other in the midline, but failed to fuse (D). White lines in A, B demonstrate the plane of section in C, D. Scale bar in A = 500 μm for A, B and 300 μm for C, D.

The skeletal analysis had demonstrated a complete cleft of the secondary palate but intact primary palate in K14-*Shh* mice. Further detailed examination using scanning electron microscopy also showed hypoplasia and dysmorphology of the upper lip in the mutants (Figs. 3A, B). In addition, the cleft region of the secondary palate was narrow, associated with close approximation of the palatal shelves and abnormal development of the surface rugae. In particular, the ante-molar rugae were lost and the inter-molar both poorly organised and diminished in size. Histological analysis from E13.5–E15.5 confirmed that the mutant palatal shelves were able to elevate above the tongue and approximate towards each other along the midline. However, in contrast to WT embryos at E15.5, there was no histological evidence of fusion in the mutant, despite considerable overlap between the shelves themselves (Figs. 3C, D). Oro-facial clefting occurs in NBCCS with an incidence of around 8.5%, which is significantly higher than that seen in the general population (Lambrecht and Kreusch, 1997; Ruprecht et al., 1987). We therefore further investigated the cleft palate phenotype of K14-*Shh* mice in an attempt to understand the embryological basis of this anomaly.

### Hedgehog signalling is increased and inappropriate in K14-*Shh* palatal shelves

*Shh* is normally expressed within the oral epithelium of the palatal shelves during early palatogenesis at E13.5, with a corresponding gradient of *Ptch1* expression in the underlying mesenchyme (Rice et al., 2006). At this stage of palatal development, Shh is known to play an important role in regulating growth and morphogenesis of the palatal shelves as they acquire a position above the tongue, prior to subsequent fusion (Rice et al., 2004; Seppala et al., 2007; Zhang et al., 2002). The epithelial expression subsequently downregulates at E14.5, becoming progressively localized to the developing rugae on the oral surface following shelf elevation, with a corresponding reduction of *Ptch1* expression in the underlying palatal mesenchyme (Rice et al., 2006).

**Fig. 4:**
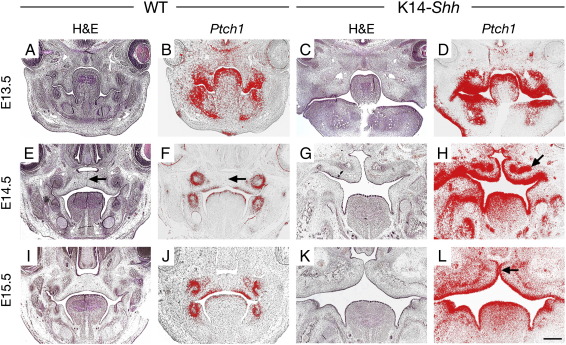
Shh signal transduction in the developing palate of WT and K14-*Shh* mice. (A, B; E, F; I, J) WT embryos from E13.5 to E15.5. A progressively reducing gradient of *Ptch1* expression was found within mesenchyme on the oral side of the palatal shelves as they elevated above the tongue and fused with their counterpart in the midline. *Ptch1* expression was absent from the MES (black arrows in E, F). (C, D; G, H; K, L) K14-*Shh* embryos from E13.5 to E15.5. *Ptch1* has a similar domain of expression to that in WT palatal shelves at E13.5. However, at E14.5 *Ptch1* was intensely expressed in the epithelium and mesenchyme (black arrow in H) of the mutant palatal shelves. Significantly, this expression remained in the epithelium of the approximating shelves at E15.5 in the region of the MES (black arrow in L). Scale bar in L = 500 μm for A–L.

We investigated the expression of *Ptch1* as a transcriptional target of Shh signalling activity in the palatal shelves of WT and K14-*Shh* mice between E13.5–E15.5 using in situ hybridisation (Figs. 4A–L). At E13.5, the domains of *Ptch1* expression were comparable between WT and K14-*Shh* mutants, indicating Shh signalling activity throughout the palatal shelf epithelium and much of the mesenchyme (Figs. 4A, B; C, D). However, between E14.5–E15.5 whilst *Ptch1* expression was seen to downregulate in WT palatal shelves (Figs. 4E, F; I, J), high levels of transcription were maintained in the mutant. In particular, *Ptch1* expression was intense within the epithelium and underlying mesenchyme of the approximating palatal shelves (Figs. 4G, H; K, L), a finding never observed at these stages in WT embryos. Together, these data suggested that increased levels of Shh signal transduction within the mutant palatal shelves was directly responsible for the cleft palate phenotype seen in these mice and a likely cause of the oro-facial clefting seen in human NBCCS.

### Palatal shelf fusion is inhibited in K14-*Shh* mice

The histological evidence implied that a defect in the process of palatal shelf fusion might be the primary cause of the cleft palate phenotype observed in K14-*Shh* embryos and this might be associated with elevated levels of Shh signal transduction in these regions. Fusion of the secondary palatal shelves is an important multi-stage component of palatogenesis and ensures mesenchymal continuity along both the midline and anterior region of the hard palate. A key feature of the fusion process is the approximation, contact and adhesion of the palatal shelf epithelia to form a midline epithelial seam (MES), which subsequently disappears in a progressive fashion via co-ordinated apoptosis (Gritli-Linde, 2007).

**Fig. 5:**
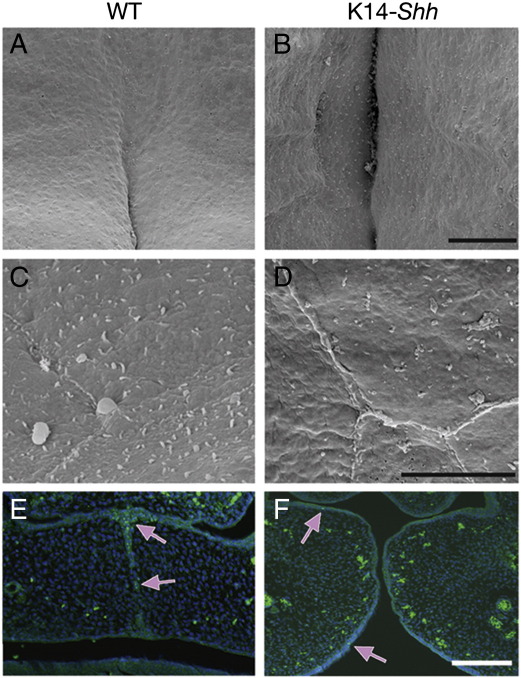
Scanning electron micrographs showing the membrane surfaces of palatal shelf oral epithelial cells in the region of the MES at E14.5. In both WT (A, C) and mutant (B, D) the intercellular junctions were clearly demarcated and numerous short filipodia present on the cell surfaces. TUNEL assay. (A) In WT shelves numerous apoptotic cells were identifiable in the regressing MES (pink arrows). (B) Mutant shelves were associated with a lack of fusion and minimal apoptosis (pink arrows) in the palatal epithelium. Scale bar in B = 100 μm for A, B; in D = 5 μm for C, D and in F = 300 μm for E, F.

Studies of palatogenesis using scanning electron microscopy have demonstrated the presence of small round cells, cellular debris, filamentous material and filopodia over the palatal shelf surface during adhesion and fusion. It has been suggested that these may represent degenerative changes in superficial cells of the MES, which facilitate subsequent adhesion (Schupbach et al., 1983). Ultrastructural examination of the oral surface of WT and K14-*Shh* palatal shelves at E14.5 in the region of the MES showed the presence of a normal and comparable epithelial cell architecture, associated with the presence of short filipodia in both WT and mutant shelves (Figs. 5A–D). However, whilst there was clear evidence of apoptosis in the regressing MES of WT embryos at the same stage, this was absent in the mutant where there was no evidence of fusion, development of a MES or any significant apoptosis within the palatal shelf epithelium (Figs. 5E, F). This provided further evidence that a failure in the normal process of fusion was taking place in the secondary palate of K14-*Shh* embryos.

**Fig. 6:**
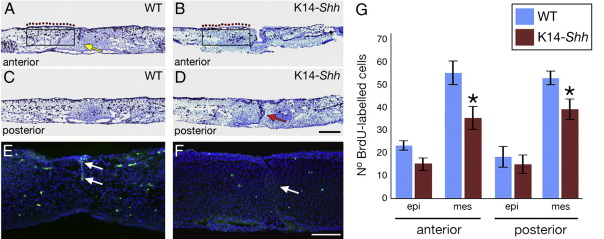
Organ culture and BrdU-labelling of palatal shelves derived from E13.5 WT and K14-*Shh* embryos. (A, C) WT palatal shelves are able to complete the process of fusion over a three day period of culture. Note regression of the MES (yellow arrow). (B, D) K14-*Shh* palatal shelves fail to fuse over three days, with persistence of the MES (red arrow). In A and B, the boxes indicate regions of mesenchymal cells counted within each palatal shelf; purple dots indicate the region of epithelial cells counted. TUNEL assay for apoptosis in cultured palatal shelves. (E) Apoptosis in the regressing MES (white arrows) of fusing WT palatal cultures. (F) In mutant palatal shelves there is no evidence of fusion or apoptosis in the medial edge epithelium (white arrow). (G) Percentage of BrdU incorporation in cultured palatal shelves. Proliferation was reduced in the epithelium and mesenchyme of both anterior and posterior K14-*Shh* palatal shelves in comparison to WT; however, this was only significant in the mesenchyme. epi, epithelium; mes, mesenchyme. Data are mean ± SD. *n* = 4 mice per group (a total of 32 WT and 28 mutant palatal shelf sections). ⁎*P *< 0.05 versus WT. Scale bar in D = 50 μm for A–D and in F = 200 μm for E, F.

It was significant that a complete approximation of the palatal shelves was never observed in the mutant embryos (see Fig. 3D) and therefore we could not rule out the possibility that this contact failure might be the primary cause of the cleft phenotype, rather than a defect in the fusion process itself. In order to further address this question we isolated palatal shelves from E13.5 WT and K14-*Shh* embryos (*n* = 4 for both groups) and cultured them in contact for 3 days prior to histological analysis (Figs. 6A–D). Those shelves derived from WT embryos were able to fuse during the period of culture, with clear evidence of apoptosis, MES degeneration and the progressive establishment of mesenchymal continuity. In contrast, mutant shelves were still unable to fuse and this was associated with persistence of the MES and no apoptosis in this region (Figs. 6E, F, arrowed). These data suggested that the normal downregulation of Shh signal transduction that is seen in the palatal shelves prior to fusion is essential for this process to take place. Interestingly, when cell proliferation was assayed in these cultured shelves, some reduction was identified in both the epithelial and mesenchymal component of the mutant, although this was only significant in the mesenchyme (Figs. 6G). Thus, the level of Shh signalling from the palatal shelf epithelium would also seem to be important in determining the levels of proliferation within the mesenchymal compartment and may have contributed to the failure of approximation that occurred in the mutant.

### Tooth development arrests at the bud stage in K14-*Shh* embryos

Odontogenic keratocysts represent the predominant anomaly found in the jaws of NBCCS subjects; however, tooth impactions and hypodontia are also seen with a significantly increased incidence compared to the general population (Manfredi et al., 2004). *Shh* is known to demonstrate a localized and specific expression pattern in the epithelial compartment of the tooth germ during early odontogenesis (Bitgood and McMahon, 1995; Cobourne et al., 2004; Hardcastle et al., 1998; Sarkar et al., 2000). In particular, being expressed in the early localized thickenings of odontogenic epithelium that demarcate the sites of tooth development and later, in the primary enamel knot; a transitory signalling centre situated within the cap stage tooth germ. These domains of expression are responsible for signalling both within the epithelial compartment of the tooth germ and from epithelium to mesenchyme; directing epithelial growth during the initiation of tooth development, establishment of the tooth bud (Cobourne et al., 2001; Hardcastle et al., 1998; Sarkar et al., 2000) and subsequent growth and morphogenesis of the tooth germ during the cap and bell stages of development (Dassule et al., 2000; Gritli-Linde et al., 2002; Jeong et al., 2004).

**Fig. 7:**
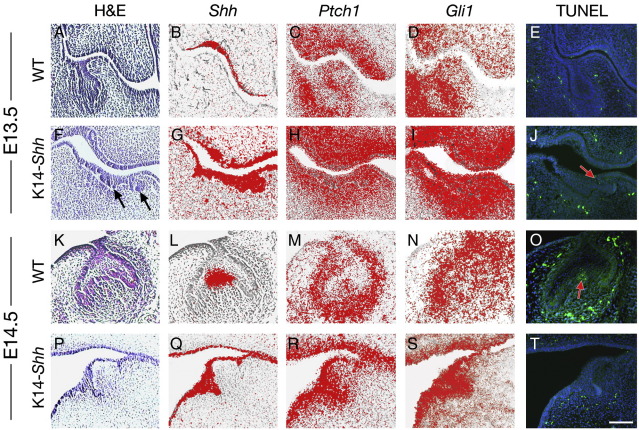
Molar tooth development in WT and K14-*Shh* mice. (A–J) At E13.5, the bud stage of development was reached in WT embryos (A) and whilst *Shh* expression was downregulated at the tip of the mature bud at this stage (B), *Ptch1* (C) and *Gli1* (D) were strongly expressed in both the epithelium and mesenchyme of the tooth germ. In mutant embryos, the teeth failed to reach the bud stage (F), with multiple shallow epithelial invaginations the only evidence of odontogenesis (black arrows). The expression of *Shh* (G) was intense in the epithelium of these teeth at this stage; whilst *Ptc1* (H) and *Gli1* (I) were strongly expressed in both the epithelium and mesenchyme. No apoptotic cells were detected in the WT bud stage tooth germ (E), although some were evident in the mutant (J, red arrow). (K–T) At E14.5, the cap stage of development had been reached in WT embryos (K) and whilst *Shh* expression was restricted to the enamel knot (L); *Ptch1* (M) and *Gli1* (N) were strongly expressed in both tissue compartments. Tooth germs failed to reach the cap stage in mutant embryos (P), the odontogenic epithelium remaining poorly organised with *Shh* (Q), *Ptch1* (R) and *Gli1* (S) very strongly expressed, particularly in the epithelial tissues. At this stage, apoptotic cells were detected in the primary enamel knot of WT tooth germs (O, red arrow) but none in the mutant (T). Scale bar in T = 50 μm for A–T.

We examined the odontogenic phenotype of WT and K14-*Shh* embryos between E13.5 and E16.5. Significantly, in mutant embryos molar tooth development failed to progress beyond a rudimentary bud stage (Figs. 7A, F; K, P). At E13.5, this was associated with either a complete absence of odontogenesis or a marked reduction in depth of the epithelial buds that were present. In many cases, multiple superficial invaginations represented the only evidence of odontogenesis that were seen, with none demonstrating any of the organisation of the bud stage invaginations observed in WT embryos (Figs. 7A, F). At E14.5, in those mutant tooth germs that had developed, some limited progression in development was seen; however, the organisation remained poor and none of these mutant molar teeth reached the well-defined cap stage seen in WT embryos (Figs. 7K, P).

These phenotypic changes were accompanied by a marked alteration in the tempero-spatial distribution of Shh signalling within the molar tooth germs. In WT embryos, *Shh* exhibits restricted and localized expression in the epithelial compartment of the tooth germ, whilst the downstream Hedgehog targets *Ptch1* and *Gli1* are expressed in both the epithelium and mesenchyme (Figs. 7B–D; L–N). In K14-*Shh* mutants, *Shh* was intensely expressed throughout the odontogenic epithelium and this was accompanied by upregulation of *Ptch1* and *Gli1* expression in both compartments, but particularly the epithelium (Figs. 7G–I; Q–S). The mutant molar tooth germs consistently failed to form a recognizable tooth bud and progress to the cap stage. This phenotype was accompanied by high-level Shh signal transduction in these teeth and suggested that regulating the level of signalling in the epithelial compartment is important during early growth and development of the tooth germ. We further investigated this arrested tooth phenotype by assaying for the presence of cell death in both WT and mutant molar tooth germs. Shh has previously been associated with the prevention of early apoptosis in the developing tooth (Cobourne et al., 2001); however, no apoptotic cells were detected in the mutant molars at either E13.5 or E14.5. This was in contrast to E14.5 WT cap stage molar tooth germs, where apoptotic cells were clearly visible in the enamel knot (Figs. 7E, J; O, T) (Vaahtokari et al., 1996).

**Fig. 8:**
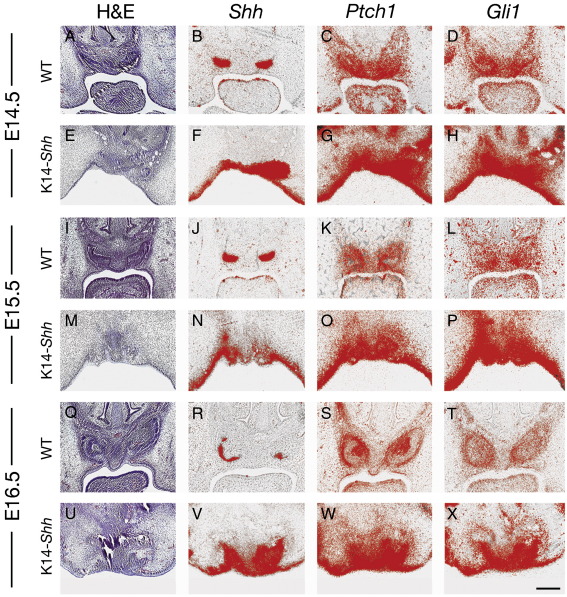
Incisor tooth development in WT and K14-*Shh* mice. (A–H) At E14.5, the cap stage of development was reached in WT embryos (A) with strong *Shh* expression in the epithelial enamel knots (B), and both *Ptch1* (C) and *Gli1* (D) in the epithelium and mesenchyme of the tooth germs. In mutant embryos, the incisor teeth failed to reach a recognizable cap stage, with multiple, poorly formed epithelial invaginations the only evidence of odontogenesis (E). There was intense *Shh* expression in the epithelial compartment of these putative teeth (F), with accompanying strong expression of *Ptch1* (G) and *Gli1* (H) in the epithelium and mesenchyme of these regions. (I–P) At E15.5, the late cap stage of development had been reached in WT embryos (I) with continued *Shh* expression restricted to the enamel knots (J) and both *Ptch1* (K) and *Gli1* (L) in odontogenic epithelium and mesenchyme. Disorganised tooth development continued in the mutant incisor tooth germs (M), along with strong epithelial expression of *Shh* (N) and both epithelial and mesenchymal expression of *Ptch1* (O) and *Gli1* (P). At E16.5, WT incisors had reached the early bell stage (Q), with reduced *Shh* expression in the epithelium (R), but continued expression of *Ptch1* (S) and *Gli1* (T) in both tissue compartments. Mutant incisor development remained disrupted, with intense expression of *Shh* (V), *Ptch1* (W) and *Gli1* (X) in this region. Scale bar in X = 50 μm for A–X.

Analysis of the mutant incisor phenotype demonstrated similar findings to those found in the molar. In contrast to WT, at E14.5 the early mutant incisor tooth germs failed to develop into clearly recognizable tooth buds. Instead, multiple localized invaginations were identifiable in the maxillary incisor region, associated with high levels of *Shh* throughout the epithelium and both *Ptch1* and *Gli1* expression in epithelium and mesenchyme. No progression of incisor tooth development was seen to take place from E14.5 through to E16.5 and this was associated with continued high levels of *Shh* transcription in these regions, in addition to *Ptch1* and *Gli1* (Figs. 8A–X).

### Arrested odontogenesis is associated with a lack of proliferation in K14-*Shh* mutant tooth germs

**Fig. 9:**
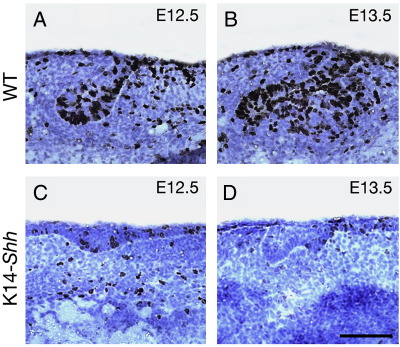
Cell proliferation in tooth germs harvested from WT and K14-*Shh* mice, and cultured for 3 days. (A) E12.5 and (B) E13.5 WT tooth germs progress to the bud and cap stages, respectively. (C) E12.5 and (D) E13.5 K14-*Shh* tooth germs failed to progress significantly beyond a rudimentary thickening or bud. BrdU-labelled cells are stained black. Scale bar in D = 50 μm for A–D.

A potential explanation for the failure of normal development in the mutant tooth germs was a lack of cellular proliferation. We investigated this by culturing molar tooth germs harvested from WT and K14-*Shh* mandibular processes at E12.5 (*n* = 3) and E13.5 (*n* = 4) for 3 days. Over the period of culture, WT tooth germs progressed to the bud and cap stages of development and this was associated with abundant cellular proliferation, particularly in the epithelial compartment (Figs. 9A, B). In contrast, mutant cultures failed to progress beyond the bud stage, those tooth germs that did form being abnormally shaped, with the majority only developing into very superficial invaginations or rudimentary buds. In all cases, this was associated with low levels of proliferating cells in the epithelial and mesenchymal tissues of the mutant teeth (Figs. 9C, D).

Together, these data show a dramatic and significant effect of increased Hedgehog signal transduction within basal epithelium on development of the craniofacial region. In particular, appropriately regulated levels of Shh are important for regulating cell fate in epithelium of the developing palate and tooth. The consequences of upregulated Shh activity form the molecular basis of the oro-facial clefting and hypodontia seen NBCCS subjects.

## Discussion

NBCCS occurs with an incidence of around 1:60,000 in Caucasians, with males and females equally affected (Manfredi et al., 2004). The vast majority of causative mutations reside in the *PTCH1* gene and inheritance is autosomal dominant, although as many as half of all cases are thought to represent new mutations (Gorlin, 1995). Most germline NBCCS mutations are truncating and concentrated in the large extracellular loops, large intracellular loop and the N-terminal region of PTCH1 (Boutet et al., 2003; Lindstrom et al., 2006; Wicking et al., 1997). However, whilst little correlation exists between the mutational type and clinical features of the NBCCS phenotype, haploinsufficiency and increased Hedgehog signal activity are thought to provide the basis of this condition (Wicking et al., 1997). The principle clinical features of NBCCS comprise BCC, odontogenic keratocysts of the jaws and skeletal anomalies; all of which can manifest in the first few decades of life (Manfredi et al., 2004). However, the diagnostic criteria are wide and up to 70% of patients will have some form of additional craniofacial anomaly. The craniofacial features associated with NBCCS can be a useful aid to early diagnosis, which is important because of the increased risk of developing juvenile carcinoma, particularly medulloblastoma. However, the aetiological basis of these developmental defects has received little attention. We therefore sought to further investigate the molecular mechanisms underlying these phenotypes using a mouse model expressing *Shh* in basal epithelium of the early face and jaws.

Hedgehog signalling is important for early craniofacial development. Targeted disruption of *Shh* in mice and mutations in human subjects can lead to holoprosencephaly [MIM #236100], which is characterized by a variable spectrum of abnormalities in the forebrain and face (Belloni et al., 1996; Chiang et al., 1996; Roessler et al., 1996). Alterations in signalling from epithelium of the early facial processes can also affect normal development. A transient loss of Shh function in the frontonasal process compromises cellular survival and proliferation, contributing to hypotelorism and clefting; whilst an increased dose can induce medio-lateral widening and hypertelorism (Hu and Helms, 1999). Moreover, inhibition of signaling using retinoic acid can truncate growth of the frontonasal and maxillary processes, producing cleft lip and palate (Helms et al., 1997). In addition, Hedgehog signalling to cranial neural crest cells is essential for the normal formation of most structures in the craniofacial skeleton, with good evidence to suggest that signal levels are crucial in the developing mouse embryo (Jeong et al., 2004). Genetically-mediated loss of signalling to cranial neural crest cells leads to facial truncation and an absence of both neural crest and non-neural crest-derived skeletal components. However, signal activation in this cell population leads to even more disruption of craniofacial development; with hyperplasia and disrupted organisation of the face, and an almost complete failure of cranial skeletal development. Significantly, these mice have a complete absence of the skull vault, including both neural crest-derived and mesodermal components, and whilst the mesodermal-derived bone absence may be secondary to increased signalling in the dorsal neural tube and consequent brain overgrowth, the direct sensitivity to signal levels in neural crest cells is clear (Jeong et al., 2004). In NBCCS, the craniofacial region is characterized by a number of skeletal anomalies; which can include frontal and parietal bossing, hypertelorism, broad nasal root, maxillary hypoplasia and mandibular prognathia. All these regions of the skull are affected in K14-*Shh* embryos and are likely to be a direct consequence of increased signalling levels within epithelial tissues. The phenotypic effects of altered signalling are complex, ocular hypertelorism has been described in mild forms of holoprosencephaly (Belloni et al., 1996), the timing of altered Hedgehog transduction during embryogenesis also significantly affects outcome (Cordero et al., 2004) and genetic modifiers can influence severity (Seppala et al., 2007).

Palatogenesis involves a series of highly co-ordinated events, with the palatine shelves of the secondary palate forming as bilateral outgrowths from the maxillary processes and initially lying adjacent to the developing tongue. These shelves have to elevate above the tongue, grow medially towards each other and fuse; both in the midline with their counterpart, anteriorly with the primary palate and superiorly with the nasal septum. The process of fusion requires co-ordinated tissue interactions, including peridermal removal, cell contact, adhesion and regression of the MES (Gritli-Linde, 2007). Any disruption in these processes of growth, elevation, or fusion will result in clefting of the secondary palate. Shh is known to contribute to early growth of murine palatal shelves through signalling from epithelium to mesenchyme, being a downstream target of Bmp2 and Fgf10/Fgf2rb signalling, and mediating both epithelial and mesenchymal cellular proliferation, which requires the induction of *Ptch1* and *Bmp2*, and function of *Gas1* (Rice et al., 2004; Seppala et al., 2007; Zhang et al., 2002). Oro-facial clefting is a recognized feature of NBCCS, occurring with a significantly increased incidence to that found in the general population (Lambrecht and Kreusch, 1997; Ruprecht et al., 1987), whilst mutations in *PTCH1* have also been associated with isolated cleft lip and palate (Mansilla et al., 2006). This suggests that a gain of Shh signalling activity might also be important in the aetiology of facial clefting. Genetic labelling has demonstrated Shh activity in localized regions of the regressing MES at E15.5 (Vaziri Sani et al., 2005); however, *Ptch1* expression is markedly downregulated in this region from E14.5 (Rice et al., 2006) (Fig. 4F, arrowed). We have shown that strong and continued *Shh* expression in the MES of K14-*Shh* mutant palatal shelves at these stages can produce clefting and that this is directly associated with an inability to fuse. This is the first report of an association between increased Shh signalling and cleft palate and provides a putative mechanism for the cleft phenotype seen in both NBCCS subjects and some cases of non-syndromic clefting. Significantly, the absence of apoptosis in epithelial cells of the mutant palatal shelf at E14.5; in contrast to the regressing MES of WT shelves, suggests that Shh might play a role in preventing cell death in these regions during the early stages of palatogenesis. In the K14-*Shh* mutant, inappropriate Hedgehog pathway activity during the process of palatal fusion might therefore prevent apoptosis in this region, leading to persistence of the MES and cleft palate. Interestingly, localized *Shh* expression is also found within the placodal regions of developing rugae on the oral surface of the palatal shelf and we have some evidence that signalling from epithelium to mesenchyme is important for subsequent development of these structures (unpublished observations). The disrupted patterning and morphogenesis of rugae seen in K14-*Shh* mice demonstrates that appropriate signal levels are also important during the development of these structures and is currently the subject of a separate investigation.

The presence of hypodontia has also been described in association with NBCCS (Manfredi et al., 2004; Ortega Garcia de Amezaga et al., 2008). Shh signalling has previously been shown to play an important role during odontogenesis; in particular, acting as a mitogen during early growth of the tooth germ (Cobourne et al., 2001; Dassule et al., 2000; Gritli-Linde et al., 2002). There is good evidence that Shh can influence the cell cycle in a number of different tissues, both embryonic and postnatal (Agathocleous et al., 2007; Roy and Ingham, 2002), and this is exemplified in NBCCS. The expression of *Shh* is highly restricted within the epithelial component of the tooth germ and it is clear that the level of activity can directly influence the epithelial cell response. This has also been demonstrated in the skin, where increasing levels of Hedgehog transgene activity can induce hyperplasia in epidermal progenitor cells up to a threshold, beyond which, negative regulation and hypoplasia takes place (Adolphe et al., 2004). Temporal regulation of Shh activity is therefore also important for epithelial cell homeostasis in the tooth germ, with the consequences of increased signalling in the epithelial component being a lack of proliferation and arrest of odontogenesis leading to hypodontia.

The oro-facial clefting and hypodontia associated with NBCCS represent secondary features, which occur in addition to the core characteristics. We have demonstrated a direct role for Shh signalling in the aetiology of these developmental defects, although in human populations they do not occur with complete penetrance. It is well known that a number of modifying factors exist that can influence the Hedgehog signalling pathway and result in considerable phenotypic variation within the same genetic background (Roessler et al., 1996; Seppala et al., 2007). These variations may well reflect altered levels of signal activity in different tissues and represent an important determinant in the manifestation of genetic disease.

## Figures and Tables

**Table 1:** Generation and analysis of K14-*Shh* transgenic embryos.

Embryonic stage	Total	Analysis			
		Skeletal preparation	SEM	Histology	Organ culture
E12.5	3⁎	0	0	0	3
	0	0	0	0	0
E13.5	9⁎	0	0	5	4
	1	0	0	1	0
E14.5	8⁎	0	3	5	0
	2	0	1	1	0
E15.5	7⁎	0	2	5	0
	2	0	0	2	0
E16.5	4⁎	0	2	2	0
	2	0	1	1	0
E17.5	3⁎	3	0	0	0
	1	1	0	0	0

A total of 42 embryos were identified as transgenic by PCR; amongst these, 34 (⁎) demonstrated severe craniofacial dysmorphology and these were the subject of the present investigation. This dysmorphology was characterized primarily by prominence of the frontal region, hypertelorism, open-bite malocclusion and cleft palate with 100% penetrance. Those transgenic embryos lacking significant craniofacial defects (*n* = 8) demonstrated varying degrees of pre-axial polysyndactyly and soft tissue syndactyly but gross morphology of the craniofacial region was normal.

## References

[bib1] Adolphe C., Narang M., Ellis T., Wicking C., Kaur P., Wainwright B. (2004). An in vivo comparative study of sonic, desert and Indian hedgehog reveals that hedgehog pathway activity regulates epidermal stem cell homeostasis. Development.

[bib2] Agathocleous M., Locker M., Harris W.A., Perron M. (2007). A general role of hedgehog in the regulation of proliferation. Cell Cycle.

[bib3] Allen B.L., Tenzen T., McMahon A.P. (2007). The Hedgehog-binding proteins Gas1 and Cdo cooperate to positively regulate Shh signaling during mouse development. Genes Dev..

[bib4] Bai C.B., Auerbach W., Lee J.S., Stephen D., Joyner A.L. (2002). Gli2, but not Gli1, is required for initial Shh signaling and ectopic activation of the Shh pathway. Development.

[bib5] Bai C.B., Stephen D., Joyner A.L. (2004). All mouse ventral spinal cord patterning by hedgehog is Gli dependent and involves an activator function of Gli3. Dev Cell..

[bib6] Belloni E., Muenke M., Roessler E., Traverso G., Siegel-Bartelt J., Frumkin A., Mitchell H.F., Donis-Keller H., Helms C., Hing A.V., Heng H.H., Koop B., Martindale D., Rommens J.M., Tsui L.C., Scherer S.W. (1996). Identification of Sonic hedgehog as a candidate gene responsible for holoprosencephaly. Nat. Genet..

[bib7] Bitgood M.J., McMahon A.P. (1995). Hedgehog and Bmp genes are coexpressed at many diverse sites of cell–cell interaction in the mouse embryo. Dev. Biol..

[bib8] Boutet N., Bignon Y.J., Drouin-Garraud V., Sarda P., Longy M., Lacombe D., Gorry P. (2003). Spectrum of PTCH1 mutations in French patients with Gorlin syndrome. J. Invest. Dermatol..

[bib9] Brunet C.L., Sharpe P.M., Ferguson M.W. (1993). The distribution of epidermal growth factor binding sites in the developing mouse palate. Int. J. Dev. Biol..

[bib10] Carstea E.D., Morris J.A., Coleman K.G., Loftus S.K., Zhang D., Cummings C., Gu J., Rosenfeld M.A., Pavan W.J., Krizman D.B., Nagle J., Polymeropoulos M.H., Sturley S.L., Ioannou Y.A., Higgins M.E., Comly M., Cooney A., Brown A., Kaneski C.R., Blanchette-Mackie E.J., Dwyer N.K., Neufeld E.B., Chang T.Y., Liscum L., Strauss J.F., Ohno K., Zeigler M., Carmi R., Sokol J., Markie D., O'Neill R.R., van Diggelen O.P., Elleder M., Patterson M.C., Brady R.O., Vanier M.T., Pentchev P.G., Tagle D.A. (1997). Niemann–Pick C1 disease gene: homology to mediators of cholesterol homeostasis. Science.

[bib11] Casali A., Struhl G. (2004). Reading the Hedgehog morphogen gradient by measuring the ratio of bound to unbound Patched protein. Nature.

[bib12] Chen Y., Struhl G. (1996). Dual roles for patched in sequestering and transducing Hedgehog. Cell.

[bib13] Chiang C., Litingtung Y., Lee E., Young K.E., Corden J.L., Westphal H., Beachy P.A. (1996). Cyclopia and defective axial patterning in mice lacking Sonic hedgehog gene function. Nature.

[bib14] Chuang P.T., McMahon A.P. (1999). Vertebrate Hedgehog signalling modulated by induction of a Hedgehog-binding protein. Nature.

[bib15] Cobourne M.T., Hardcastle Z., Sharpe P.T. (2001). Sonic hedgehog regulates epithelial proliferation and cell survival in the developing tooth germ. J. Dent. Res..

[bib16] Cobourne M.T., Miletich I., Sharpe P.T. (2004). Restriction of sonic hedgehog signalling during early tooth development. Development.

[bib17] Corbit K.C., Aanstad P., Singla V., Norman A.R., Stainier D.Y., Reiter J.F. (2005). Vertebrate Smoothened functions at the primary cilium. Nature.

[bib18] Cordero D., Marcucio R., Hu D., Gaffield W., Tapadia M., Helms J.A. (2004). Temporal perturbations in sonic hedgehog signaling elicit the spectrum of holoprosencephaly phenotypes. J. Clin. Invest..

[bib19] Dassule H.R., Lewis P., Bei M., Maas R., McMahon A.P. (2000). Sonic hedgehog regulates growth and morphogenesis of the tooth. Development.

[bib20] Davies J.P., Chen F.W., Ioannou Y.A. (2000). Transmembrane molecular pump activity of Niemann–Pick C1 protein. Science.

[bib21] Dessaud E., Yang L.L., Hill K., Cox B., Ulloa F., Ribeiro A., Mynett A., Novitch B.G., Briscoe J. (2007). Interpretation of the sonic hedgehog morphogen gradient by a temporal adaptation mechanism. Nature.

[bib22] Evans D.G., Ladusans E.J., Rimmer S., Burnell L.D., Thakker N., Farndon P.A. (1993). Complications of the naevoid basal cell carcinoma syndrome: results of a population based study. J. Med. Genet..

[bib23] Goodrich L.V., Johnson R.L., Milenkovic L., McMahon J.A., Scott M.P. (1996). Conservation of the hedgehog/patched signaling pathway from flies to mice: induction of a mouse patched gene by Hedgehog. Genes Dev..

[bib24] Goodrich L.V., Milenkovic L., Higgins K.M., Scott M.P. (1997). Altered neural cell fates and medulloblastoma in mouse patched mutants. Science.

[bib25] Gorlin R.J. (1995). Nevoid basal cell carcinoma syndrome. Dermatol. Clin..

[bib26] Gorlin R.J., Goltz R.W. (1960). Multiple nevoid basal-cell epithelioma, jaw cysts and bifid rib. A syndrome. N. Engl. J. Med..

[bib27] Gritli-Linde A. (2007). Molecular control of secondary palate development. Dev. Biol..

[bib28] Gritli-Linde A., Bei M., Maas R., Zhang X.M., Linde A., McMahon A.P. (2002). Shh signalling within the dental epithelium is necessary for cell proliferation, growth and polarization. Development.

[bib29] Hahn H., Wicking C., Zaphiropoulous P.G., Gailani M.R., Shanley S., Chidambaram A., Vorechovsky I., Holmberg E., Unden A.B., Gillies S., Negus K., Smyth I., Pressman C., Leffell D.J., Gerrard B., Goldstein A.M., Dean M., Toftgard R., Chenevix-Trench G., Wainwright B., Bale A.E. (1996). Mutations of the human homolog of *Drosophila* patched in the nevoid basal cell carcinoma syndrome. Cell.

[bib30] Hardcastle Z., Mo R., Hui C.C., Sharpe P.T. (1998). The Shh signalling pathway in tooth development: defects in Gli2 and Gli3 mutants. Development.

[bib31] Haycraft C.J., Banizs B., Aydin-Son Y., Zhang Q., Michaud E.J., Yoder B.K. (2005). Gli2 and Gli3 localize to cilia and require the intraflagellar transport protein polaris for processing and function. PLoS Genet..

[bib32] Helms J.A., Kim C.H., Hu D., Minkoff R., Thaller C., Eichele G. (1997). Sonic hedgehog participates in craniofacial morphogenesis and is down-regulated by teratogenic doses of retinoic acid. Dev. Biol..

[bib33] Hu D., Helms J.A. (1999). The role of sonic hedgehog in normal and abnormal craniofacial morphogenesis. Development.

[bib34] Huangfu D., Anderson K.V. (2005). Cilia and Hedgehog responsiveness in the mouse. Proc. Natl. Acad. Sci. U. S. A..

[bib35] Huangfu D., Liu A., Rakeman A.S., Murcia N.S., Niswander L., Anderson K.V. (2003). Hedgehog signalling in the mouse requires intraflagellar transport proteins. Nature.

[bib36] Ingham P.W., McMahon A.P. (2001). Hedgehog signaling in animal development: paradigms and principles. Genes Dev..

[bib37] Jeong J., Mao J., Tenzen T., Kottmann A.H., McMahon A.P. (2004). Hedgehog signaling in the neural crest cells regulates the patterning and growth of facial primordia. Genes Dev..

[bib38] Johnson R.L., Rothman A.L., Xie J., Goodrich L.V., Bare J.W., Bonifas J.M., Quinn A.G., Myers R.M., Cox D.R., Epstein E.H., Scott M.P. (1996). Human homolog of patched, a candidate gene for the basal cell nevus syndrome. Science.

[bib39] Kimonis V.E., Goldstein A.M., Pastakia B., Yang M.L., Kase R., DiGiovanna J.J., Bale A.E., Bale S.J. (1997). Clinical manifestations in 105 persons with nevoid basal cell carcinoma syndrome. Am. J. Med. Genet..

[bib40] Lambrecht J.T., Kreusch T. (1997). Examine your orofacial cleft patients for Gorlin–Goltz syndrome. Cleft Palate-Craniofac. J..

[bib41] Lindstrom E., Shimokawa T., Toftgard R., Zaphiropoulos P.G. (2006). PTCH mutations: distribution and analyses. Hum. Mutat..

[bib42] Liu A., Wang B., Niswander L.A. (2005). Mouse intraflagellar transport proteins regulate both the activator and repressor functions of Gli transcription factors. Development.

[bib43] Loftus S.K., Morris J.A., Carstea E.D., Gu J.Z., Cummings C., Brown A., Ellison J., Ohno K., Rosenfeld M.A., Tagle D.A., Pentchev P.G., Pavan W.J. (1997). Murine model of Niemann–Pick C disease: mutation in a cholesterol homeostasis gene. Science.

[bib44] Manfredi M., Vescovi P., Bonanini M., Porter S. (2004). Nevoid basal cell carcinoma syndrome: a review of the literature. Int. J. Oral Maxillofac. Surg..

[bib45] Mansilla M.A., Cooper M.E., Goldstein T., Castilla E.E., Lopez Camelo J.S., Marazita M.L., Murray J.C. (2006). Contributions of PTCH gene variants to isolated cleft lip and palate. Cleft Palate-Craniofac. J..

[bib46] Martin V., Carrillo G., Torroja C., Guerrero I. (2001). The sterol-sensing domain of Patched protein seems to control Smoothened activity through Patched vesicular trafficking. Curr. Biol..

[bib47] Martinelli D.C., Fan C.M. (2007). Gas1 extends the range of Hedgehog action by facilitating its signaling. Genes Dev..

[bib48] May S.R., Ashique A.M., Karlen M., Wang B., Shen Y., Zarbalis K., Reiter J., Ericson J., Peterson A.S. (2005). Loss of the retrograde motor for IFT disrupts localization of Smo to cilia and prevents the expression of both activator and repressor functions of Gli. Dev. Biol..

[bib49] McMahon A.P., Ingham P., Tabin C. (2003). Developmental roles and clinical significance of hedgehog signalling. Curr. Top. Dev. Biol..

[bib50] Mo R., Freer A.M., Zinyk D.L., Crackower M.A., Michaud J., Heng H.H., Chik K.W., Shi X.M., Tsui L.C., Cheng S.H., Joyner A.L., Hui C. (1997). Specific and redundant functions of Gli2 and Gli3 zinc finger genes in skeletal patterning and development. Development.

[bib51] Oro A.E., Higgins K.M., Hu Z., Bonifas J.M., Epstein E.H., Scott M.P. (1997). Basal cell carcinomas in mice overexpressing sonic hedgehog. Science.

[bib52] Ortega Garcia de Amezaga A., Garcia Arregui O., Zepeda Nuno S., Acha Sagredo A., Aguirre Urizar J.M. (2008). Gorlin–Goltz syndrome: clinicopathologic aspects. Med. Oral Patol. Oral Cir. Bucal..

[bib53] Pan Y., Bai C.B., Joyner A.L., Wang B. (2006). Sonic hedgehog signaling regulates Gli2 transcriptional activity by suppressing its processing and degradation. Mol. Cell Biol..

[bib54] Park H.L., Bai C., Platt K.A., Matise M.P., Beeghly A., Hui C.C., Nakashima M., Joyner A.L. (2000). Mouse Gli1 mutants are viable but have defects in SHH signaling in combination with a Gli2 mutation. Development.

[bib55] Rice R., Spencer-Dene B., Connor E.C., Gritli-Linde A., McMahon A.P., Dickson C., Thesleff I., Rice D.P. (2004). Disruption of Fgf10/Fgfr2b-coordinated epithelial–mesenchymal interactions causes cleft palate. J. Clin. Invest..

[bib56] Rice R., Connor E., Rice D.P. (2006). Expression patterns of Hedgehog signalling pathway members during mouse palate development. Gene Expr. Patterns.

[bib57] Roessler E., Belloni E., Gaudenz K., Jay P., Berta P., Scherer S.W., Tsui L.C., Muenke M. (1996). Mutations in the human Sonic Hedgehog gene cause holoprosencephaly. Nat. Genet..

[bib58] Rohatgi R., Milenkovic L., Scott M.P. (2007). Patched1 regulates hedgehog signaling at the primary cilium. Science.

[bib59] Rossi A., Caracciolo V., Russo G., Reiss K., Giordano A. (2008). Medulloblastoma: from molecular pathology to therapy. Clin. Cancer Res..

[bib60] Roy S., Ingham P.W. (2002). Hedgehogs tryst with the cell cycle. J. Cell Sci..

[bib61] Ruprecht A., Austermann K.H., Umstadt H. (1987). Cleft lip and palate, seldom seen features of the Gorlin–Goltz syndrome. Dentomaxillofac. Radiol..

[bib62] Sarkar L., Cobourne M., Naylor S., Smalley M., Dale T., Sharpe P.T. (2000). Wnt/Shh interactions regulate ectodermal boundary formation during mammalian tooth development. Proc. Natl. Acad. Sci. U. S. A..

[bib63] Schupbach P.M., Chamberlain J.G., Schroeder H.E. (1983). Development of the secondary palate in the rat: a scanning electron microscopic study. J. Craniofac. Genet. Dev. Biol..

[bib64] Seppala M., Depew M.J., Martinelli D.C., Fan C.M., Sharpe P.T., Cobourne M.T. (2007). Gas1 is a modifier for holoprosencephaly and genetically interacts with sonic hedgehog. J. Clin. Invest..

[bib65] Shanley S., Ratcliffe J., Hockey A., Haan E., Oley C., Ravine D., Martin N., Wicking C., Chenevix-Trench G. (1994). Nevoid basal cell carcinoma syndrome: review of 118 affected individuals. Am. J. Med. Genet..

[bib66] Stamataki D., Ulloa F., Tsoni S.V., Mynett A., Briscoe J. (2005). A gradient of Gli activity mediates graded Sonic Hedgehog signaling in the neural tube. Genes Dev..

[bib67] Stone D.M., Hynes M., Armanini M., Swanson T.A., Gu Q., Johnson R.L., Scott M.P., Pennica D., Goddard A., Phillips H., Noll M., Hooper J.E., de Sauvage F., Rosenthal A. (1996). The tumour-suppressor gene patched encodes a candidate receptor for Sonic hedgehog. Nature.

[bib68] Strutt H., Thomas C., Nakano Y., Stark D., Neave B., Taylor A.M., Ingham P.W. (2001). Mutations in the sterol-sensing domain of Patched suggest a role for vesicular trafficking in Smoothened regulation. Curr. Biol..

[bib69] Taipale J., Cooper M.K., Maiti T., Beachy P.A. (2002). Patched acts catalytically to suppress the activity of Smoothened. Nature.

[bib70] Tempe D., Casas M., Karaz S., Blanchet-Tournier M.F., Concordet J.P. (2006). Multisite protein kinase A and glycogen synthase kinase 3beta phosphorylation leads to Gli3 ubiquitination by SCFbetaTrCP. Mol. Cell. Biol..

[bib71] Tenzen T., Allen B.L., Cole F., Kang J.S., Krauss R.S., McMahon A.P. (2006). The cell surface membrane proteins Cdo and Boc are components and targets of the Hedgehog signaling pathway and feedback network in mice. Dev. Cell.

[bib72] Vaahtokari A., Aberg T., Thesleff I. (1996). Apoptosis in the developing tooth: association with an embryonic signaling center and suppression by EGF and FGF-4. Development.

[bib73] Vassar R., Rosenberg M., Ross S., Tyner A., Fuchs E. (1989). Tissue-specific and differentiation-specific expression of a human K14 keratin gene in transgenic mice. Proc. Natl. Acad. Sci. U. S. A..

[bib74] Vaziri Sani F., Hallberg K., Harfe B.D., McMahon A.P., Linde A., Gritli-Linde A. (2005). Fate-mapping of the epithelial seam during palatal fusion rules out epithelial–mesenchymal transformation. Dev. Biol..

[bib75] Wang B., Li Y. (2006). Evidence for the direct involvement of {beta}TrCP in Gli3 protein processing. Proc. Natl. Acad. Sci. U. S. A..

[bib76] Wicking C., Shanley S., Smyth I., Gillies S., Negus K., Graham S., Suthers G., Haites N., Edwards M., Wainwright B., Chenevix-Trench G. (1997). Most germ-line mutations in the nevoid basal cell carcinoma syndrome lead to a premature termination of the PATCHED protein, and no genotype–phenotype correlations are evident. Am. J. Hum. Genet..

[bib77] Zhang X.M., Ramalho-Santos M., McMahon A.P. (2001). Smoothened mutants reveal redundant roles for Shh and Ihh signaling including regulation of L/R asymmetry by the mouse node. Cell.

[bib78] Zhang Z., Song Y., Zhao X., Zhang X., Fermin C., Chen Y. (2002). Rescue of cleft palate in Msx1-deficient mice by transgenic Bmp4 reveals a network of BMP and Shh signaling in the regulation of mammalian palatogenesis. Development.

